# Methylprednisolone versus Dexamethasone for Control of Vertigo in Patients with Definite Meniere's disease

**Published:** 2017-11

**Authors:** Elham Masoumi, Sasan Dabiri, Mohammad Taghi Khorsandi Ashtiani, Reza Erfanian, Saeed Sohrabpour, Nasrin Yazdani, Alireza Safaee, Mohammadreza Firouzifar

**Affiliations:** 1 *Otorhinolaryngology Research Center, Tehran University of Medical Sciences, Tehran, Iran.*

**Keywords:** Dexamethasone, Intratympanic injection, Meniere disease, Methylprednisolone, Vertigo

## Abstract

**Introduction:**

Definite Meniere's disease is associated with two or more definitive periods of vertigo along with hearing loss, plus tinnitus or aural fullness or both. This study aimed to compare the effect of intratympanic dexamethasone and methylprednisolone on the functional-level scale of pure-tone audiometry (PTA), and class outcome measures of vertigo.

**Materials and Methods:**

In this clinical study, 69 patients with definite Meniere's disease, referred to the tertiary otolaryngology center, were randomly assigned to two groups: 36 patients were treated with intratympanic dexamethasone (4mg/dl) and 33 patients were treated with i*ntratympanic* *methylprednisolone (40mg/dl). Each group received three weekly injections. After a follow-up of 1 and 6* months, PTA changes and vertigo control were evaluated.

**Results:**

There was no statistically significant difference between the two groups with regard to control of vertigo (P=0.866, P=0.879 for 1 and 6 months post injection, respectively). PTA improvement was statistically significantly higher in the methylprednisolone group (P=0.006).

**Conclusion:**

In summary, intratympanic corticosteroid is an effective treatment for Meniere's disease and can prevent other invasive treatments. Intratympanic methylprednisolone can improve hearing level to a greater extent than intratympanic dexamethasone, but the two groups were similarly beneficial in controlling vertigo. However, there was a trend toward a more sustained benefit with methylprednisolone.

## Introduction

Meniere's disease (MD) is an inner-ear disease manifesting with symptoms of frequent vertigo (96.2%), tinnitus (91.1%), sensorineural hearing loss (87.7%), and aural fullness, which occurs because of endolymphatic hydrops (1). The prevalence of MD varies internationally; for example, 10.7 in 100,000 individuals in Japan and 513 per 100,000 people in Finland have been reported to suffer from MD (1). This may be because of different diagnostic criteria or follow-up periods (2). The pathogenesis of the disease is endolymphatic hydrops, and the most plausible theory is insufficient absorption of endolymph by the endolymphatic sac (1).

There is no single diagnostic test for the diagnosis of MD, and its best definition is based on the criteria of the American Academy of Otolaryngology–Head and Neck Surgery (*AAO*–*HNS*). The *AAO–HNS* criteria for the diagnosis of MD include vertigo, hearing loss and tinnitus. On this basis, MD is divided into possible, probable, definite and certain forms (1). MD symptoms such as vertigo, drop attacks, tinnitus, aural fullness and hearing loss can severely affect the patient’s quality of life (3). The available treatment options are lifestyle modification (salt and caffeine intake limitation and adequate hydration), medical therapy (primarily consisting of diuretics and injections of intratympanic [IT] corticosteroids or gentamicin), de*compression* *of* *the endolymphatic* *sac,* and vestibular nerve section (4). The use of topical steroids (IT injection), allowing the appropriate cumulative dose in the desired tissues without systemic use of the steroids and associated side effects, seems logical (5). Due to the ototoxicity risks, many therapists prefer to use an IT corticosteroid injection instead of gentamicin (6). According to the current literature, IT dexamethasone injections can safely improve MD symptoms (vertigo and hearing loss but not tinnitus) and the patient’s quality of life (2,7-12), although some studies report that this effect is transitory (9,13,14) Methylprednisolone can result in higher concentrations of steroid in the *perilymphatic fluid*. 

On the other hand, studies have shown that mineralocorticoid receptor binding has an important role in the improvement of hearing, and methylprednisolone has a greater binding affinity for this receptor than dexamethasone (15). In one study, the use of IT methylprednisolone was shown to achieve a 90% reduction in vertigo attacks in intractable MD (16).

To the best of our knowledge, there are no data on whether different corticosteroids differ in terms of MD management. In this study, we compared the results of IT injections of dexamethasone and methylprednisolone in MD patients based on a functional-level scale of audiometry and a class outcome measure of vertigo.

## Materials and Methods

Eighty patients with a definite MD diagnosis according *AAO*–*HNS criteria* (refractory to treatment with salt restriction, diuretics, and betahistine for 3 months) referred to Amir Alam Hospital between July 2015 and December 2016 were enrolled in this study after providing written consent. Inclusion criteria were age greater than 18 years, no history of other otologic diseases, normal magnetic resonance imaging (MRI) scan and no history of neurological disorders. Patients with middle-ear infections, conductive hearing loss, history of ototoxic drug usage, history of ear surgery (*ossiculoplasty* and *stapedotomy*) and inner-ear surgery, *bilateral* *MD*; perforated tympanic membrane, addiction, spinal cord disease,* diseases of the central nervous system* (*CNS*), neuromuscular diseases, and pregnancy were excluded. Cerebellopontine (CP) angle lesions were ruled out pre-study using MRI. Patients with a prior history of glucocorticoid intake 1 month before enrolling into the trial and during the trial were also excluded.

*Ethical considerations* *:*

Patients were informed about the study and provided signed informed consent. Patients voluntarily participated in the study and were free to leave the study at their will. Possible drug and treatment side effects were fully disclosed at the beginning of study.

*Intervention method* *:*

Eighty patients were randomly assigned to two groups using block randomization (N1=N2=40). 

Group 1 received IT dexamethasone (4mg/dl) and Group 2 received IT methylprednisolone (40mg/dl) three times a week. Four patients in Group 1 and seven in Group 2 were lost to follow up. At data cut-off, 36 patients in Group 1 and 33 patients in Group 2 were analyzed in this study. A questionnaire requesting the name, age, sex, chief complaint (hearing loss, vertigo, or tinnitus), time of onset, duration of the disease, associated symptoms, and other comorbid disorders was completed by the patients. In this questionnaire, pure-tone audiometry (PTA) results, including the frequency specific threshold and the presence or absence of stapedius reflex and tympanogram, were recorded. Hearing level was analyzed using AAO–HNS criteria (Table.1), while the vertigo numeric scale value was determined based on AAO–HNS criteria (Table.2).

**Table 1:** AAO-HNS criteria for hearing level assessment.

Stage	Four-Tone Average (dB)
1	≤25
2	26-40
3	41-70
4	>70

a. Hearing is measured using a four-frequency pure-tone average (PTA) of 500HZ, 1KHZ, 2KHZ, and 3KHZ

b. Pretreatment hearing level: worst hearing level during 6 months before surgery

c. Post-treatment hearing level: Poorest hearing level measured 18-24 months after institution of therapy

d. Hearing classification:

i. Unchanged: ≤10-dB PTA improvement or worsening or ≤15% speech discrimination improvement or worsening

ii. Improved: >10-dB PTA improvement or >15% discrimination improvement

iii. Worse: >10-dB PTA worsening or >15% discrimination worsening

In 1996, the Committee on Hearing and Equilibrium reaffirmed and clarified the guidelines, adding initial staging and reporting guidelines:

**Table 2:** Vertigo numeric scale value

**Numeric value**	**Control level**	**Class**
0–40	Complete control of definitive spells	A
41–80	Limited control of definitive spells	B
81–120	Insignificant control of definitive spells	C

To undergo the procedure, patients were asked to adopt the supine position. Lidocaine was used to induce local anesthesia. In both groups, agents were injected into the anterior inferior part of the tympanic membrane until the middle ear was filled. Patients remained in the supine position for 15 minutes and were instructed not to swallow for the first few minutes after injection. Three injections were performed within a week. Four weeks after the last injection, the audiometry test was repeated and then the patients entered the follow-up period. Six months after the last injection, patients underwent the audiometry test again, and the rate of improvement of vertigo was evaluated using the class outcome measure of vertigo. Patients received no other treatments during the follow-up period.

*Statistical analysis* *:*

Data were analyzed using SPSS 20.0. The Friedman test was used to compare the mean difference in vertigo. Then, a 2×2 Wilcoxon signed rank test was used before the intervention, as well as 1- and 6-months post intervention. A PTA comparison for both groups was performed using the Kolmogorov–Smirnov test. A p-value less than 0.05 was considered statistically signficant. As the data distribution was non-normal, nonparametric statistics were used.

## Results

Demographic data are presented in Table 3. Before the intervention, there was no significant difference in hearing levels between the two groups (Table.4). However, there was a significant difference in hearing level between the two groups 6 months after intervention (Table.5).

**Table 3:** Patient demographic data

			P
**Age** ** (years** **)**	39.9 ± 13.84	41.51 ± 11.68	0.588
**Sex ** **(** **Male** **; ** **Female** **)**	18 M; 15 F	21M; 15 F	0.470
**Side ** **(right** **;** **left)**	22R; 14L	16R; 17L	0.339
**Duration **	4.5455±1.98	4.0556±3.12	0.444

**Table 4:** Hearing level before intervention

**Group**	**Hearing level**			**P-value**
	I	II	III	
Dexamethasone	20	11	5	0.604
Methylprednisolone	15	15	3	

**Table 5:** Hearing levels after intervention

**Groups**	**Hearing level ** **after intervention**			**P-value**
	Improved	Unchanged	Worse	
Methylprednisolone (intratympanic)	18	13	2	0.006*
Dexamethasone	4	32	0	
Total	22	45	2	

* significant difference

Vertigo was recorded based on AAO–HNS criteria (Table.2) at three cut-off points:

before, 1 month and 6 months after the last injection. Results are presented in Table 6. Within-group comparisons revealed significant differences in vertigo between pre-treatment and 1 month after the last injection (P<0.001), between pre-treatment and 6 months after the last injection (P=0.002), and between 1 month and 6 months after the last injection (P=0.001) in the methylprednisolone group and in vertigo between pre-treatment and 1 month after the last injection (P<0.001) and between 1 month and 6 months after the last injection (P<0.001) in the dexamethasone group. 

There was no significant difference in vertigo between pre-treatment and 6 months after the last injection in the dexamethasone group (P=0.206) (Table.7).

**Table 6:** Vertigo, before, one and six months after intervention between the two groups

**Variable**	**Study ** **group**						**Total**
	**Group A**		**Group B**		**Group C**		p-value
	MP	Dex	MP	Dex	MP	Dex	
Vertigo before intervention	0	0	20	27	13	9	0.301
Vertigo after 1 month	18	21	11	12	4	3	0.866
Vertigo after 6 month	7	6	17	19	9	11	0.879

MP, Methylprednisolone; Dex, Dexamethasone

**Table 7:** Within-group analysis of vertigo before, 1 and 6 months after intervention

		**Vertigo before–** **1 month after**	**Vertigo before–** **6 months after**	**Vertigo 1 month–** **6 months after**
Dex	Z Sig.	−5.196 ^*^0.000	−1.265 0.206	−4.413 *0.000
MP	Z Sig.	−4.354 *0.000	−3.051 *0.002	−3.234 *0.001

Dex: Dexamethasone; MP: Methylprednisolone

* significant difference

## Discussion

To the best of our knowledge, this is the first study to compare the effects of two corticosteroids in the treatment of MD for vertigo control and hearing changes.

In this study, the rates of vertigo control in at least one category on a numeric scale 1 month after treatment were 75% and 66% for the dexamethasone and methylprednisolone groups, respectively. Therefore, it seems initially that the efficacy of dexamethasone is higher. However, after 6 months, treatment efficacy reduced in both groups. In both groups, vertigo control declined in the 6 months after the last treatment. However, while vertigo control returned to the pre-treatment level in the dexamethasone group after 6 months, it remained higher than the pre-treatment level in the methylprednisolone group. In an inter-group analysis, the two groups were not significantly different.

The results of this study differed from the results of other studies. In a study conducted by She in 2015, in which the effectiveness of methylprednisolone in the treatment of patients with drug-*resistant MD was assessed*, the level of vertigo control in the first 6 months after the intervention and in the prolonged follow-up period were 94% and 81%, respectively. This difference is probably due to the different ways in which the projects were conducted (16). In the She study, after diagnosis and confirmation of intractable *MD* among the patients, they were hospitalized for 10 days. Also, after inserting the IT catheter 20mg/0.5ml daily for 10 days, the patients were treated with methylprednisolone through the same catheter. According to this study, in the 2 hours after the injection, the maximum concentration of methylprednisolone is created in the perilymphatic fluid, and its effect persists up to 6 hours, before reducing gradually over the 24 hours after the injection. For the same reason, for maximum effectiveness of the drug, injections were performed on a daily basis and patients underwent intravenous treatment daily with different drugs such as Ginkgo or derivatives of vitamin B12. The follow-up of patients was 2 to 3 years, which differed substantially from the current study.

In another study conducted by Patel in 2016 (30 patients), a reduction of 90% in vertigo was reported in patients in the methylprednisolone group in the first 6 months after treatment with *IT* *methylprednisolone*. In this study, the dose of *IT* *methylprednisolone* was 62.5mg/ml, administered as two injections with an interval of 2 weeks. In addition, these patients entered a pre-treatment phase for 6 months before enrolling in the study. Moreover, the patients were compared in the 6 months before and 6 months after the intervention, and the duration of follow-up was 2 years (15).

In the present study, hearing was improved both quantitatively and qualitatively in the two groups, although the difference was not significant in the dexamethasone group; similar to other studies. We also found a significant difference between the two groups regarding hearing improvement. A number of review articles support our results suggesting a role for IT steroids on vertigo and hearing level improvement in MD (17-19), but no articles are available that compare different steroids in this matter.

In a cohort study by Gabra et al. in 2013, 89 patients with a diagnosis of MD were enrolled, of whom 47 patients were treated with IT gentamicin (ITG) and 42 were treated with methylprednisolone (ITMP). The patients underwent follow-up in two episodes, and control of attacks of vertigo, tinnitus, ear fullness stages, PTA, and SDS were examined from baseline to 6 months and from 6 to 12 months. Between 6 and 12 months after the injection in the ITG group and the ITMP group, an improvement of 82.9% and 48.1%, respectively, in vertigo was reported. In addition, the control of tinnitus and ear fullness was better in the ITG group compared with the ITMP group, and in both groups there was no difference in terms of hearing before and after the injection (20). This seems contrary to our results in that we found hearing improvement and better vertigo control in our patients with methylprednisolone. Furthermore, a Cochrane review has suggested that gentamicin as a vestibulotoxic drug has a detrimental effect on hearing (21), so the results of this article should be interpreted with caution.

*Limitations* Limitations of this study include the small sample size and limited follow-up. Also in this study, there was no pre-treatment phase and a single- or double-blind method was not used.

*Recommendations*

Conducting a study with a wider size and longer follow-up is recommended. In addition, a microcatheter insertion procedure and daily injections may prove good alternatives to the periodic IT injection. Other audiologic and objective measurements such as electrocochle- ography and vestibular evoked myogenic potential may also be compared between groups to better delineate possible differences.

## Conclusion

  According to this study, IT corticosteroid may be an interesting and non-invasive method of decreasing MD symptoms. Both of these agents showed a temporary effect on vertigo control. IT methylprednisolone achieves better hearing level improvement than IT dexamethasone, and may also have a more sustained efficacy in controlling vertigo in MD patients.
